# 3D dSTORM imaging reveals novel detail of ryanodine receptor localization in rat cardiac myocytes

**DOI:** 10.1113/JP277360

**Published:** 2018-11-28

**Authors:** Xin Shen, Jonas van den Brink, Yufeng Hou, Dylan Colli, Christopher Le, Terje R. Kolstad, Niall MacQuaide, Cathrine R. Carlson, Peter M. Kekenes‐Huskey, Andrew G. Edwards, Christian Soeller, William E. Louch

**Affiliations:** ^1^ Institute for Experimental Medical Research Oslo University Hospital and University of Oslo NO‐0424 Oslo Norway; ^2^ Simula Research Laboratory 1364 Fornebu Norway; ^3^ Department of Chemistry University of Kentucky Lexington KY USA; ^4^ Institute of Cardiovascular Sciences University of Glasgow Glasgow UK; ^5^ Living Systems Institute University of Exeter Exeter UK; ^6^ KG Jebsen Center for Cardiac Research University of Oslo Oslo Norway

**Keywords:** 3D super‐resolution imaging, Ryanodine Receptors, calcium homeostasis, excitation‐contraction coupling, t‐tubule

## Abstract

**Key points:**

- Using 3D direct stochastic optical reconstruction microscopy (dSTORM), we developed novel approaches to quantitatively describe the nanoscale, 3D organization of ryanodine receptors (RyRs) in cardiomyocytes.
- Complex arrangements of RyR clusters were observed in 3D space, both at the cell surface and within the cell interior, with allocation to dyadic and non‐dyadic pools.
- 3D imaging importantly allowed discernment of clusters overlapping in the *z*‐axis, for which detection was obscured by conventional 2D imaging techniques. Thus, RyR clusters were found to be significantly smaller than previous 2D estimates.
- Ca^2+^ release units (CRUs), i.e. functional groupings of neighbouring RyR clusters, were similarly observed to be smaller than earlier reports. Internal CRUs contained more RyRs in more clusters than CRUs on the cell surface, and yielded longer duration Ca^2+^ sparks.

**Abstract:**

Cardiomyocyte contraction is dependent on Ca^2+^ release from ryanodine receptors (RyRs). However, the precise localization of RyRs remains unknown, due to shortcomings of imaging techniques which are diffraction limited or restricted to 2D. We aimed to determine the 3D nanoscale organization of RyRs in rat cardiomyocytes by employing direct stochastic optical reconstruction microscopy (dSTORM) with phase ramp technology. Initial observations at the cell surface showed an undulating organization of RyR clusters, resulting in their frequent overlap in the *z*‐axis and obscured detection by 2D techniques. Non‐overlapping clusters were imaged to create a calibration curve for estimating RyR number based on recorded fluorescence blinks. Employing this method at the cell surface and interior revealed smaller RyR clusters than 2D estimates, as erroneous merging of axially aligned RyRs was circumvented. Functional groupings of RyR clusters (Ca^2+^ release units, CRUs), contained an average of 18 and 23 RyRs at the surface and interior, respectively, although half of all CRUs contained only a single ‘rogue’ RyR. Internal CRUs were more tightly packed along *z*‐lines than surface CRUs, contained larger and more numerous RyR clusters, and constituted ∼75% of the roughly 1 million RyRs present in an average cardiomyocyte. This complex internal 3D geometry was underscored by correlative imaging of RyRs and t‐tubules, which enabled quantification of dyadic and non‐dyadic RyR populations. Mirroring differences in CRU size and complexity, Ca^2+^ sparks originating from internal CRUs were of longer duration than those at the surface. These data provide novel, nanoscale insight into RyR organization and function across cardiomyocytes.

## Key points

- Using 3D direct stochastic optical reconstruction microscopy (dSTORM), we developed novel approaches to quantitatively describe the nanoscale, 3D organization of ryanodine receptors (RyRs) in cardiomyocytes.
- Complex arrangements of RyR clusters were observed in 3D space, both at the cell surface and within the cell interior, with allocation to dyadic and non‐dyadic pools.
- 3D imaging importantly allowed discernment of clusters overlapping in the *z*‐axis, for which detection was obscured by conventional 2D imaging techniques. Thus, RyR clusters were found to be significantly smaller than previous 2D estimates.
- Ca^2+^ release units (CRUs), i.e. functional groupings of neighbouring RyR clusters, were similarly observed to be smaller than earlier reports. Internal CRUs contained more RyRs in more clusters than CRUs on the cell surface, and yielded longer duration Ca^2+^ sparks.

## Introduction

In cardiac myocytes, ryanodine receptors (RyRs) are integral membrane proteins localized on the sarcoplasmic reticulum (SR). With dimensions of approximately 29 × 29 × 12 nm, these tetrameric proteins are located in dyadic junctions with the surface sarcolemma and transverse‐tubules (t‐tubules) (Franzini‐Armstrong & Protasi, 1997; Franzini‐Armstrong *et al*. 1999). During excitation–contraction coupling, influx of Ca^2+^ via dyadic L‐type calcium channels triggers opening of apposed RyRs, and a much greater release of Ca^2+^ from the SR. Precise understanding of this process requires detailed knowledge of the spatial arrangement of these Ca^2+^‐handling proteins. The large and electron‐dense structure of the RyR has enabled direct visualization by electron microscopy (EM), and early studies suggested that the protein intrinsically forms tightly packed, crystalline‐like clustered arrays (Franzini‐Armstrong & Protasi, 1997; Yin & Lai, 2000). However, more recent work utilizing EM tomography and 2D super‐resolution imaging has shown that dyads are unlikely to be completely filled with RyRs, and that dyads often contain multiple RyR clusters (Baddeley *et al*. 2009; Hayashi *et al*. 2009; Jayasinghe *et al*. 2018). Moreover, these studies have indicated that Ca^2+^ diffusion distances between neighbouring RyR clusters are often sufficiently short (<100 nm) to support their concerted generation of Ca^2+^ sparks (Sobie *et al*. 2006), the fundamental Ca^2+^ release events in myocytes (Cannell *et al*. 1995). Termed ‘Ca^2+^ release units’ (CRUs), these functional arrangements of RyRs are suggested to have dynamic organization (Asghari *et al*. 2014), which is crucial in determining the characteristics of Ca^2+^ sparks (Walker *et al*. 2014; Macquaide *et al*. 2015).

Despite continued improvements in high resolution imaging modalities, the true 3D localization of key dyadic proteins such as the RyR remains unknown, data that are critical for creating accurate computational models of excitation–contraction coupling (Hake *et al*. 2014; Walker *et al*. 2014). Indeed, previous work using 2D direct stochastic optical reconstruction microscopy (dSTORM) has often been limited to the cell surface, where the localization of RyRs within the SR has been assumed to be relatively flat to enable grid‐based protein counting (Baddeley *et al*. 2009). Visualization of internal RyR clusters, on the other hand, has been hampered by expected overlapping fluorescence from out‐of‐plane RyRs, particular in the *z*‐ (axial) plane where resolution is typically more modest (Hou *et al*. 2015). To circumvent these limitations, we have presently developed a novel, quantitative 3D dSTORM method based on phase ramp imaging localization microscopy (PRILM) (Baddeley *et al*. 2011 *a*), to investigate the 3D organization of RyRs in rat ventricular myocytes. We show that both surface and interior RyR clusters exhibit a complex arrangement in 3D space, with surface clusters exhibiting particularly disordered organization. By comparison, we observed that 2D imaging approaches employed in previous work significantly overestimate the sizes of both RyR clusters and CRUs. We further show that combining RyR dSTORM imaging with 3D images of t‐tubules enables direct visualization and quantification of 3D RyR geometry within dyads, and comparison with non‐dyadic RyRs. Revealed differences in CRU morphology at the cell surface and interior were linked to differences in Ca^2+^ spark morphology observed at these sites.

## Methods

### Ethical approval

All animal protocols were performed in accordance with the Norwegian Animal Welfare Act and NIH Guidelines and were approved by the Ethics Committee of the University of Oslo.  Experiments were performed on adult male Wistar rats (250–350 g) purchased from Janvier Labs (Le Genest‐Saint‐Isle, France). Rats were group housed with *ad libitum* access to food and water, and maintained at 22°C on a 12 h:12 h light–dark cycle. A total of 15 animals were employed. The authors understand the ethical principles under which *The Journal of Physiology* operates and declare that our work complies with its animal ethics checklist.

### Rat ventricular cardiomyocyte isolation and preparation for imaging

Cell isolation was based on the protocol described by Hodne *et al*. (2017). Animals were anaesthetized with isoflurane and sacrificed via cervical dislocation. Hearts were quickly excised, cannulated, and mounted on a constant‐flow (3 ml min^−1^), Langendorff set‐up. The heart was first perfused with Ca^2+^‐free oxygenated solution (in mmol l^−1^: NaCl 140, KCl 5.4, MgCl_2_ 0.5, NaH_2_PO_4_ 0.4, Hepes 5, glucose 5.5, pH 7.4), and then switched to collagenase type II (2 mg ml^−1^, Worthington Biochemical Corp., Lakewood, NJ, USA) containing solution for 10–12 min at 37 °C. Following digestion, left ventricular tissue was dissected and diced into 3–4 mm^3^ pieces. In order to free additional cells from tissue, a secondary digestion was performed by transferring approximately 8 ml of tissue and collagenase solution to a 10 ml Falcon tube containing 0.2 mg DNase (LS002006, Worthington) in 500 μl BSA. Cells were then filtered, allowed to pellet, and extracellular [Ca^2+^]_o_ gradually increased to 1 mmol l^−1^.

In most experiments, longitudinal imaging of cardiomyocytes was enabled by plating cells on glass bottom dishes (No. 1.5, Ø 14 mm, γ‐irradiated, MatTek Corp., Ashland, MA, USA) that had been coated with laminin (mouse, BD Biosciences, San Jose, CA, USA) overnight at 4°C. The plated cells were washed twice with Dulbecco's phosphate‐buffered saline (DPBS; No. 4387, BioWhittaker Inc., Walkersville, MD, USA), fixed with 4% paraformaldehyde (PFA) for 10 min, quenched with 100 μmol l^−1^ glycine for 10 min, permeabilized with 1% Triton X‐100 for 10 min, and finally blocked using a high blocking buffer (5% goat serum, 3% BSA and 0.02% NaN_3_ in DPBS) for 2 h at room temperature.

In a subset of experiments, isolated cardiomyocytes were prepared for imaging in the transverse orientation. PFA‐fixed cells were first embedded in optimal cutting temperature (OCT) compound (Fisher Scientific, Gothenburg, Sweden), then snap frozen with liquid nitrogen. The frozen block was reoriented perpendicularly, to stand cardiomyocytes on end, and subsequently cut into 10 μm‐thick cryosections using a Cryostar NX70 cryostat (Thermo Fisher Scientific, Oslo, Norway). Sections were plated together with intact, fixed isolated cells in 35 mm glass bottom imaging dishes (P35G‐1.5‐14‐C, MatTek Corp.).

### Immunofluorescence labelling

For RyR labelling, cells were incubated with mouse anti‐RyR (1:100, MA3‐916, Thermo Fisher Scientific) overnight at 4 °C. For t‐tubule visualization, cells were incubated overnight at 4°C in a mixture of rabbit anti‐Cav‐3 (1:100, ab2912, Abcam, Cambridge, UK) antibody and a custom rabbit anti‐NCX1 antibody (1:100, Genscript Corp., Piscataway, NJ, USA, described below). A similar antibody cocktail approach has been previously shown to effectively label t‐tubules (Jayasinghe *et al*. 2012). Secondary antibody labelling was performed with donkey anti‐mouse Alexa Fluor 647 (1:200, ab150103, Abcam) and goat anti‐rabbit CF 568 (1:200, 20099, VWR International, Oslo, Norway) antibodies for 2 h at room temperature. These pre‐adsorbed, fab‐fragment secondary antibodies place the fluorescent labels far closer to the epitope than traditional antibody labels. Consequently, the steric error from indirect antibody labelling is generally <10 nm both laterally and axially under our experimental conditions, and largely negligible in comparison with the calculated localization precision. Both primary and secondary antibodies were diluted in a low blocking buffer (2% goat serum, 1% BSA and 0.02% NaN_3_ in DPBS).

The custom rabbit polyclonal NCX antibody was created with core sequence EYDDKQPLTSKEEEERRI, and specificity verified by epitope mapping. The cytosolic loop (amino acids 243–799) of rat NCX1 protein (EDM02743) was synthesized as 20‐mer peptides with three‐amino acid offsets on cellulose membranes using a Multipep automated peptide synthesizer (Intavis Bioanalytical Instruments AG, Cologne, Germany). The membranes were further activated in methanol for 10 s, washed 3 × 10 min in Tris‐buffered Saline and Tween 20 (TBS‐T) and blocked in 1% casein for 1 h at room temperature. The membranes were then incubated with or without anti‐NCX1 (no. 3299, core epitope: EYDDKQPLTSKEEEERRI; GenScript, NJ, USA) overnight at 4 °C with gentle agitation, washed three times for 10 min in TBS‐T and incubated with a horseradish peroxidase‐conjugated secondary antibody. Blots were developed using ECL Prime (RPN 2232, GE Healthcare). The chemiluminescence signals were detected by a Las‐4000 (Fujifilm, Tokyo, Japan).

### 3D dSTORM super resolution imaging of RyRs

AlexaFluor 647‐labelled rat ventricular cardiomyocytes were mounted in VectaShield (H‐1000, Vector Laboratories, Burlingame, CA, USA), a medium which has been shown to produce similar, if not superior quality dSTORM images when compared with conventional oxygen scavenging based systems (Olivier *et al*. 2013). Cells were imaged using the Zeiss ELYRA system coupled to an LSM 710 microscope (Carl Zeiss, Jena, Germany). Briefly, a 150 mW diode 642 nm laser was focused onto the sample via a plan‐apo ×100, 1.46 NA oil objective in a highly inclined and laminated optical sheet (HiLo). 3D imaging was enabled via the modification of the point spread function (PSF) using PRILM technology (Baddeley *et al*. 2011 *a*), where a double phase ramp is inserted into the pupil plane of the back aperture of the objective. Emitted fluorescence >655 nm was collected with a iXon 897 back‐thinned EMCCD camera (Andor Technology, Belfast, UK). A sequence of 20,000 frames was acquired for each cell at a frame exposure time of 50 ms. During image acquisition, a piezo‐operated definite focus system was utilized to autocorrect for axial drift.

### 3D dSTORM image reconstruction and analysis

Reconstruction of 3D dSTORM data was conducted using the proprietary ‘PALM Processing’ module in the ZEN software (Zeiss). Briefly, a 3D experimental PSF was calculated across an axial range of 4 μm using 100 nm TetraSpecks (T7279, Thermo Fisher Scientific). Single molecule events were detected with a 19 pixel circular mask size, with a signal to background noise ratio of 6. Each localization event was assigned to a points table with a corresponding *x*, *y* and *z* coordinate. To correct for lateral drift, a five piece‐wise linear function was applied to localization events. In order to minimize the inclusion of clusters with larger localization error, events from only the central 600 nm of the 4 μm stack were included (Baddeley *et al*. 2011 *a*). This was sufficient to visualize potential RyR clusters that may be associated with an entire t‐tubule in rat cardiomyocytes (Soeller & Cannell, 1999).

Image reconstruction, rescaling, and thresholding were performed using a custom‐written script in Python. For image reconstruction, individual events were fitted with a Gaussian function which corresponded to the precision value (in nanometres). This was outputted as a 600 nm‐thick stack with a voxel size of 10 nm. For quantitative cluster analysis, the voxel size was scaled down to 30 × 30 × 30 nm such that each voxel will at most contain only a solitary RyR. Otsu‐based automated thresholding was then applied to binarize the images. Finally, the script measured the number of events from a given voxel, which was later used to determine the number of events per RyR cluster.

### Estimation of surface and interior RyR numbers

Calculation of RyR distribution between peripheral and interior sites was based on labelling densities measured by dSTORM, and an idealized cardiomyocyte shape with the dimensions of an elliptic cylinder: volume ≈ 25,000 μm^3^ (Bensley *et al*. 2016), short axis radius = 6 μm, long axis radius = 10 μm, length = 130 μm. Based on cross‐sectional dSTORM images (Fig. 2 *E*), interior RyR clusters were assumed to be excluded from the outermost 1.5 μm of the idealized geometry.

### Confocal imaging of RyRs and t‐tubules (NCX1/Cav‐3) for correlative reconstruction of dSTORM images

The Zeiss ELYRA LSM 710 microscope employed for dSTORM imaging was also used for confocal imaging of RyRs and t‐tubules for 3D correlative reconstruction. This approach eliminated the need to transfer samples and relocate cells between microscopes. CF 568‐labelled t‐tubules and AF 647‐labelled RyRs were imaged using laser lines 561 nm and 633 nm, respectively. 3D image stacks were obtained with a frame size of 1600 × 1600 pixels and a *z*‐spacing of 200 nm per slice. Deconvolution was conducted with Huygens Essential software, with a signal to noise ratio of 5.0. Other steps for image analysis were performed in ImageJ unless otherwise stated.

Widefield mode was initially used to visually inspect labelling of RyRs and t‐tubules, and select a region of interest for which the focal position was noted. Switching to confocal mode, a 3 μm *z*‐stack of RyRs and t‐tubules was acquired which was vertically centred on this focal position. The microscope was then switched to dSTORM mode, and a 20,000 frame 3D sequence of the previously determined region was acquired with matched focus. Confocal stacks were deconvolved, and dSTORM images were reconstructed as appropriate. The pixel size of the confocal image was resized to match that of the dSTORM image (10 × 10 × 10 nm in *x*, *y* and *z*, respectively). The alignment of the two sets of images was done by comparing confocal and dSTORM recordings of RyRs from the same area of interest, employing a 2D point‐based approach using the periphery of the cell as a fiduciary landmark (Crossman *et al*. 2015). A custom‐written script corrected for both translational and scaling differences between the two imaging modalities, including correction for shifts in the axial direction.

### 3D geometric rendering of dSTORM RyRs and confocal t‐tubules

The 3D arrangement of RyRs within dyads was estimated by examining points of interface between t‐tubules, determined by confocal NCX1/Cav‐3 images, and dSTORM‐based RyR positions within junctional SR (jSR). A geometry was constructed by first estimating t‐tubular structures using a custom script in Python. t‐tubule *z*‐stack data were thresholded using a global mean histogram threshold, short components not contiguous with the rest of the network were pruned (volume <0.03 μm³), and the resulting binarized structures were skeletonized.

Morphological dilatation of the skeleton was then performed using a spherical structuring element to produce uniformly cylindrical t‐tubules 250 nm in diameter, in agreement with literature estimates for rat cardiomyocytes (Soeller & Cannell, 1999). A boundary for the adjoining jSR mask was set by slight further dilatation of the t‐tubule elements to produce a 10 nm dyadic cleft. The dSTORM‐identified RyR cluster positions were then overlaid with the reconstructed t‐tubules. The majority of the clusters directly overlapped with the jSR mask, defining an interface area. These clusters were deemed to be dyadic, and the interface area was filled with RyRs using an RyR–RyR centre distance of 30 nm. Remaining dSTORM‐identified RyR clusters that did not directly overlap with the jSR mask were found at a wide range of distances from any t‐tubule. Due to limited precision in the confocal imaging as well as inherent inaccuracies in the skeletonization and re‐dilatation the t‐tubule geometries, clusters lying within 250 nm of a t‐tubule were also deemed to be dyadic and projected onto the nearest jSR mask area. A similar distance threshold for distinction of dyadic and non‐dyadic RyRs has been employed in previous work (Jayasinghe *et al*. 2009). Non‐dyadic cluster sizes were estimated with a calibration curve created using dyadic clusters, correlating the number of events to RyR number.

To produce the junctional SR terminals for each cluster, the fitted RyRs were iteratively dilated using a cross‐shaped structuring element to produce a jSR padding of 80 nm around each RyR, limited to the jSR mask defined earlier. Finally, to visualize the resulting geometry, iso‐surfaces for the t‐tubules, jSR terminals and individual RyRs were generated from the voxel‐based geometries using the Marching‐Cubes algorithm. The iso‐surfaces were then smoothed using Geometry‐preserving Adaptive MeshER (GAMer) (Lorensen & Cline, 1987; Yu *et al*. 2008) and finally rendered using Blender (Blender Foundation, Amsterdam, Netherlands). A representative 3D geometry was animated with assistance from Fluks AS (Oslo, Norway).

### Ca^2+^ Spark imaging and analysis

To gain insight into the functional consequences of RyR organization, Ca^2+^ sparks were recorded in isolated cardiomyocytes loaded with fluo‐4 AM (20 μm, Thermo Fisher Scientific) and superfused with Hepes Tyrode solution containing (in mmol l^−1^): 140 NaCl, 1.8 CaCl_2_, 0.5 MgCl2, 5.0 Hepes, 5.5 glucose, 0.4 NaH_2_PO_4_ and 5.4 KCl (pH 7.4, 37°C). Using an LSM 7 Live microscope (Zeiss), a 1024 × 50 pixel (163.6 × 8 μm) imaging frame was selected near the *z*‐axis centre of the cell, which included both the cell surface and the interior. A total of 1000 frames was recorded at a rate of 1.85 ms per frame. For analysis, sparks were analysed with custom software, implemented in Python 3.6.5 utilizing JupyterLab, NumPy, SciPy's ndimage multi‐dimensional image processing library, and the Scikit‐Image library. Confocal microscopic images were denoised and segmented as described by Gonzalez & Woods (1992). Sparks were identified via *ad hoc* thresholding of Sobel filter responses applied in the time dimension. Sparks were fit with an exponential growth function to determine full duration at half maximum (FDHM) and fit with a two‐dimensional Gaussian function to determine full width at half maximum (FWHM). Spark proximity to the sarcolemma was identified using spark centroid and distance transforms of segmented myocytes. The routine used for spark detection and analysis is available at https://bitbucket.org/Dcolli23/spark_analysis/.

### Statistical analyses

Values presented are means ± SEM unless otherwise stated. Differences between sample means were tested with either paired or unpaired Student's *t*‐test or nested ANOVA, as appropriate. *P* values <0.05 were considered to be significant.

## Results

### Comparison of surface RyR clusters in 2D and 3D

**Figure 1:**
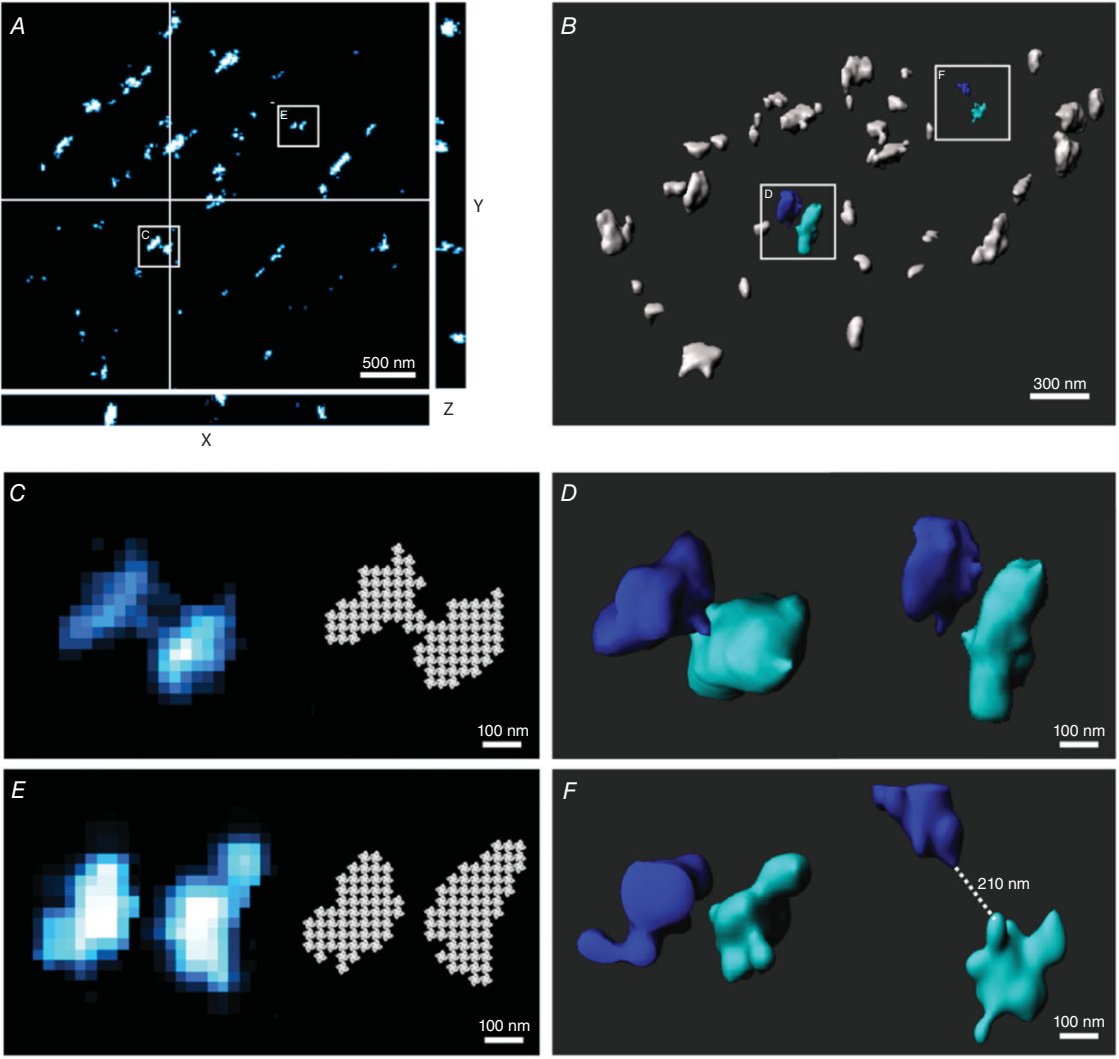
3D dSTORM imaging of RyRs at the surface of isolated rat cardiomyocytes Orthogonal (*A*) and 3D (*B*) views of RyRs at the cell surface illustrating their undulating, non‐horizontal organization, with variable localization in the axial plane. Zoomed images of the indicated regions are provided in the lower panels, in both 2D (*C* and *E*) and 3D (*D* and *F*). 2D imaging resulted in erroneous merging of vertically aligned RyR clusters and inaccurate quantification using grid‐based methods for RyR counting (*C*). However, 3D imaging showed such clusters to be distinct (*D*). Clusters which were not axially overlapping were nevertheless observed to have underestimated inter‐cluster spacing in 2D relative to 3D imaging (*E* and *F*, respectively).

Previous attempts to quantify cardiomyocyte RyR localization by 2D super‐resolution imaging have enabled RyR counting by fitting thresholded images to a 30 × 30 nm mask, corresponding to the dimensions of the RyR tetramer (Baddeley *et al*. 2009). By restricting such methods to the surface of the cells, it has been assumed that the RyR clusters have a relatively flat arrangement, without overlap in the axial (*z*) axis. However, when we currently applied PRILM‐based 3D dSTORM imaging at the cell surface, we observed an undulating, somewhat irregular arrangement of RyR clusters in this plane (representative orthogonal and 3D views shown in Fig. 1 *A* and *B*, respectively). This non‐horizontal configuration resulted in overlap of some neighbouring RyR clusters in the *z*‐axis, as illustrated in Fig. 1 *C* and *D*. In this example, nearby clusters observed to be distinct in 3D imaging appear contiguous when projected in 2D. Thus, thresholding and grid‐based counting of 2D images (Fig. 1 *C*, right panel) is expected to underestimate the number of RyR clusters at the cell surface, while overestimating their size. In cases where neighbouring clusters did not overlap in the axial plane, 2D imaging was nevertheless observed to frequently underestimate the true spacing between clusters in 3D (Fig. 1 *E* and *F*).

**Figure 2:**
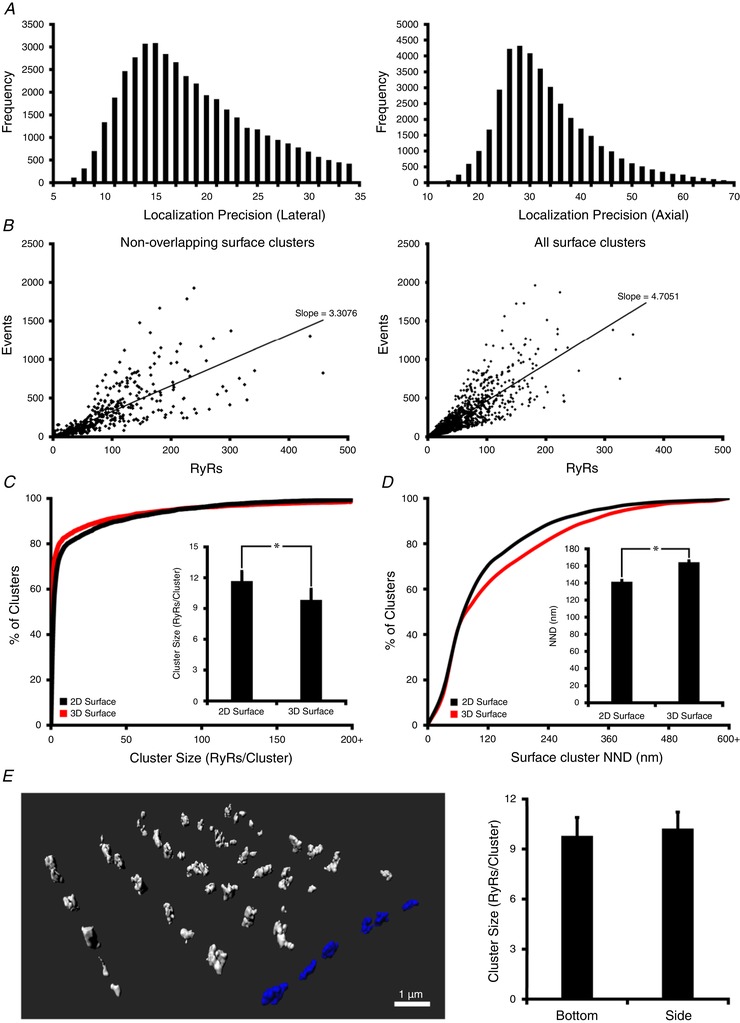
Quantification 3D RyR cluster characteristics at the cell surface *A*, lateral (left panel) and axial (right panel) localization precision of recorded RyR events (blinks). *B*, a linear relationship was observed between the number of events and the number of RyRs estimated by grid‐based counting (see Fig. 1 *C* and *E*). A significantly less steep correlation was observed when measurements were restricted to non‐overlapping surface clusters (left panel) rather than all surface clusters (right panel), as merging of vertically aligned clusters was circumvented. The slope of this curve was used as a calibration factor to estimate the number of RyRs within clusters in 3D images. *C*, 3D quantification revealed smaller RyR clusters than obtained by 2D projections, as indicated by a cumulative percentage histogram and mean measurements (inset), and a higher proportion of single‐cluster RyRs (56% *vs*. 35% of all clusters). *D*, in agreement with representative images shown in Fig. 1 *E* and *F*, 2D imaging also underestimated the distance between neighbouring clusters (nearest‐neighbour distance; NND), as indicated by a cumulative percentage histogram and mean data (inset, *n*_cells_ = 15, *n*_hearts_ = 4). *E*, To verify the validity of our calibration technique with varying axial plane, we compared the characteristics of 3D‐imaged RyR clusters located on the side of the cell (shown blue in 3D schematic representation) with those located on the bottom of the cell. Employing the calibration factor to recordings at the two surface locations revealed identical cluster sizes (right panel; *n*_cells_ = 9, *n*_hearts_ = 3). ^*^ *P* < 0.05 *vs*. 2D measurements.

While these findings suggest that 3D imaging provides an opportunity for more accurate assessment of RyR cluster geometry, quantification of cluster sizes in 3D is more complex than with 2D approaches. Due to the properties of the point‐spread function (PSF), localization precision is lower in the axial (*z*) direction than within the lateral (*xy*) plane (Fig. 2 *A*). Thus, the mask‐fitting technique employed for 2D images (Fig. 1 *C* and *E*, right panels) cannot simply be extended to a cuboid mask approach in 3D without introducing error. We instead approached the quantification of 3D RyRs by creating a calibration curve, with correlation of the number of fluorescence events (blinks) to the number of RyRs (Fig. 2 *B*). This curve was created by selecting surface RyR clusters which were observed to be non‐overlapping in 3D, and then projecting these images in 2D to enable RyR counting by the conventional masking method. Plotting and correlating these values revealed a best‐fit line with slope = 3.3076 events/RyR (*R* ^2^ = 0.729, from 1740 clusters, Fig. 2 *B* left panel), a value which can then be used to estimate the number of RyRs within any 3D‐imaged cluster. Of note, this calibration curve was less steep than one created assuming all surface clusters are flat (Fig. 2 *B*, right panel), supporting that ‘merging’ of vertically overlapping RyR clusters causes overestimation of cluster sizes when conventional 2D approaches are employed. Indeed, estimating RyR counts based on the localization of blinks in 3D revealed a significantly lower number of RyRs/cluster in comparison with 2D quantification (10.1 ± 1.1 *vs*. 11.8 ± 1.0 RyRs/clusters, *P* < 0.05, Fig. 2 *C*). This included a leftward shift of the cumulative percentage histogram toward an increased proportion of small RyRs, and identification of more single, isolated RyRs (56.0% *vs*. 34.8% of all clusters). As implied by images of neighbouring clusters (Fig. 1 *E* and *F*), 3D imaging also revealed greater inter‐cluster spacing than 2D imaging; a cumulative percentage histogram of nearest‐neighbour distance (NND) was right‐shifted and mean measurements were increased (164 ± 2 *vs*. 141 ± 2 nm, *P* < 0.05, Fig. 2 *D*). These results suggest that previous work employing 2D dSTORM imaging is likely to have overestimated the size of RyR clusters at the cell surface, while underestimating their spacing (Baddeley *et al*. 2009).

To corroborate the accuracy of our calibration method, we compared the geometries of RyR clusters located on the bottom of the cell closest to the coverslip (as above) with clusters located on the sides of the cell. While RyR cluster sizes would be expected to be similar at these two surface locations, limitations in the axial resolution could complicate quantification. Despite these challenges, 3D imaging and application of the calibration method revealed nearly identical cluster sizes on the sides and bottom of the cell (Fig. 2 *E*). This finding suggests that 3D dSTORM with suitable calibration as shown here can be employed to accurately discern and quantify RyR clusters even in the axial plane.

### Quantification of Ca^2+^ release units

**Figure 3:**
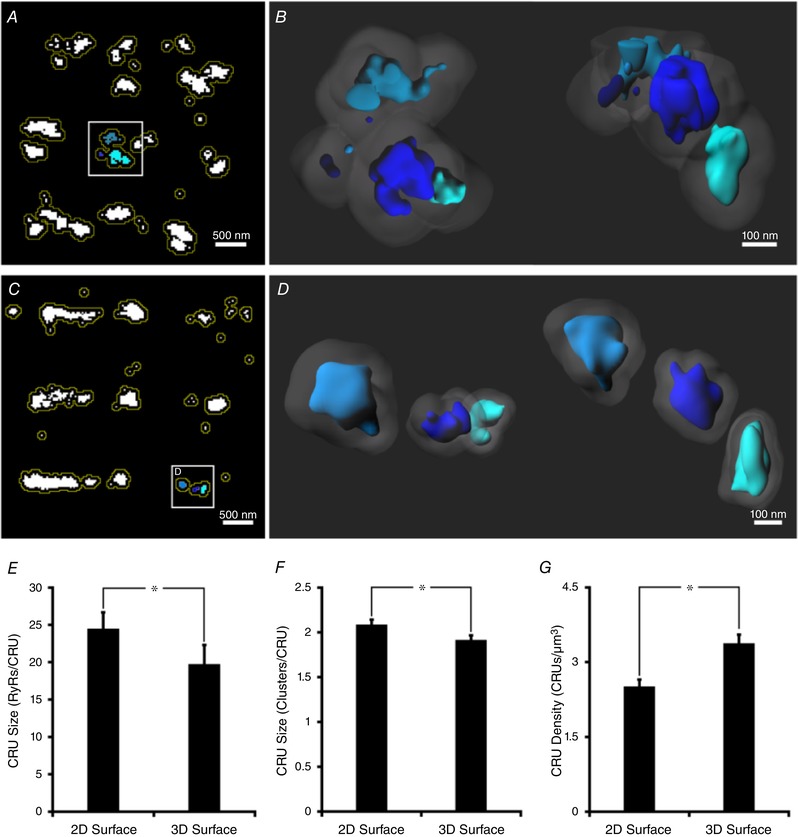
Quantitative comparison of cell surface Ca^2+^ release units in 2D and 3D *A*, in 2D images, Ca^2+^ release units (CRUs) were defined by grouping RyR clusters with lateral edge‐to‐edge distances within 100 nm. This was accomplished by dilating clusters by 50 nm (yellow lines), and collecting those which fused. *B*, for 3D images, CRU inclusion criteria also incorporated the axial plane, as shown schematically for the indicated CRU in *A*, with both top‐down and side‐on views (left and right panels, respectively; 50 nm cluster dilatations shown shaded). *C*, 2D imaging resulted in erroneous grouping of clusters into CRUs. An example grouping of 3 RyR clusters shown in the inset was observed to constitute 3 distinct CRUs when imaged in 3D (*D*, top‐down and side views), as inter‐cluster distances were >100 nm. With improved accuracy of CRU definition, 3D imaging revealed smaller mean CRU sizes, with fewer contained RyRs (*E*) and clusters (*F*) than estimated by 2D approaches, and higher average CRU density (*G*). *n*_cells_ = 15, *n*_hearts_ = 4; ^*^ *P* < 0.05 *vs*. 2D measurements.

Recent work based on mathematical modelling has indicated that closely localized RyR clusters, with edge‐to‐edge distances within 100 nm, may act cooperatively to generate Ca^2+^ sparks (Sobie *et al*. 2006). These functional RyR groupings are referred to as superclusters or Ca^2+^ release units (CRUs; Baddeley *et al*. 2009), and can only be defined by precise nanoscale imaging. We investigated whether 3D dSTORM imaging provides novel insight into CRU geometry. To quantify CRUs, individual clusters were laterally dilated by 50 nm in 2D images (Fig. 3 *A*), and by 50 nm in all three dimensions in 3D (Fig. 3 *B*). Dilated regions were subsequently fused, and all clusters contained within each newly formed region were identified as members of the same CRU.

More accurate assessment of inter‐cluster distances by 3D dSTORM frequently resulted in fewer clusters being included in a given CRU in comparison with 2D‐based estimates. An example is shown in Fig. 3 *C*, where 2D imaging suggested that three neighbouring clusters were localized within 100 nm of each other, and should be grouped into the same CRU. 3D imaging, however, revealed that the true nearest‐neighbour distances between these clusters were greater than 100 nm, meaning that each cluster should be defined as being its own CRU (Fig. 3 *D*). Indeed, overall CRU size estimates were significantly lower when 3D analysis was employed, in terms of both the number of included RyRs (17.5 ± 1.3 *vs*. 22.6 ± 1.2 in 2D, Fig. 3 *E*) and clusters (1.9 ± 0.1 *vs*. 2.1 ± 0.1 in 2D, Fig. 3 *F*). With fewer clusters in each CRU, 3D analysis revealed a higher density of surface CRUs than provided by 2D estimates (3.4 ± 0.2 *vs*. 2.5 ± 0.1 CRUs μm^−3^, *P* < 0.05, Fig. 3 *G*).

### Comparison of surface and interior RyR clusters in 3D

**Figure 4:**
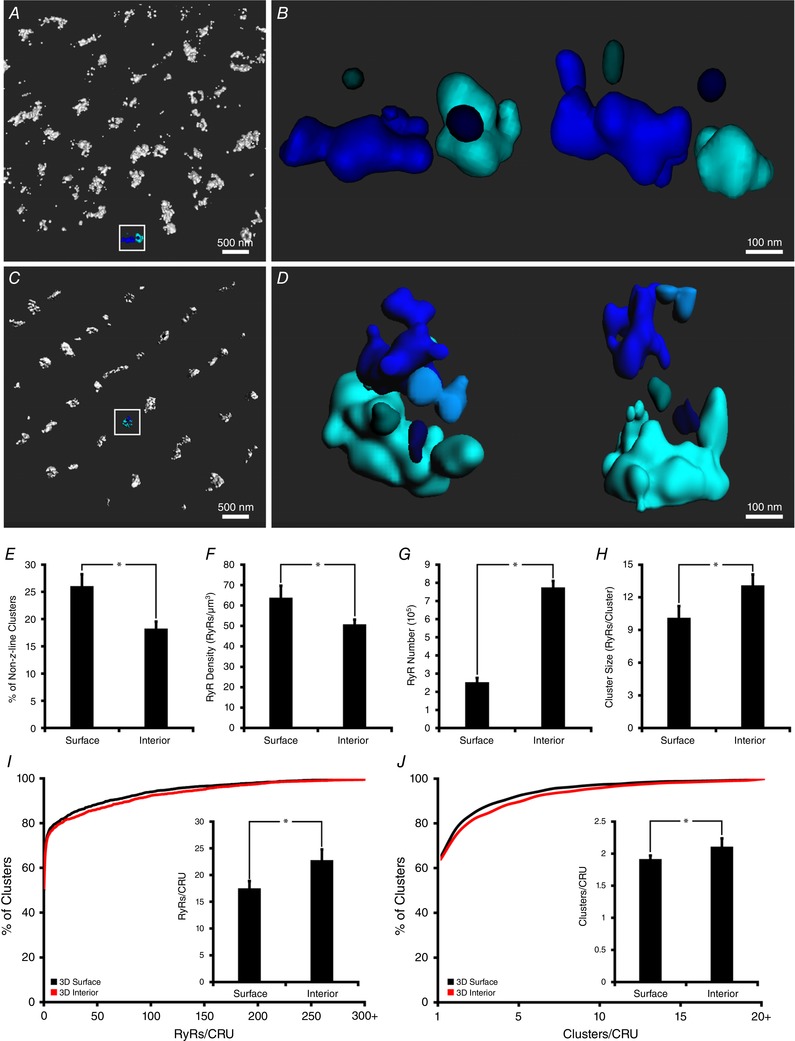
Comparison of 3D RyR organization at the cell surface and interior Representative 3D renderings of RyR clusters at the cell surface and interior are illustrated in *A* and *C*, respectively. Greater detail of example CRUs from the indicated regions are shown in *B* and *D*, with top down (left) and side‐on (right) views. In comparison with RyRs localized at the cell surface, internal sites exhibited fewer clusters localized between *z*‐lines (*E*), and lower overall RyR density (*F*). However, since internal sites occupy a greater fraction of the cell volume, the majority (∼75%) of the cell's ∼1,000,000 RyRs were localized within the cell interior (*G*). Tighter packing of RyRs along *z*‐lines at internal sites was associated with larger cluster sizes (*H*), and larger CRUs, as defined by the number of contained RyRs (*I*, cumulative percentage histogram with mean values in inset) and clusters (*J*). *n*_cells_ = 15, *n*_hearts_ = 4 for both surface and internal images. ^*^ *P* < 0.05 *vs*. surface measurements. [Corrections added on 14 December 2018, after first online publication: *A* represents the cell surface and *C* represents the interior]

The 3D organization of RyRs at internal sites in cardiomyocytes is expected to be complex, with significant vertical alignment of RyR clusters proximal to t‐tubules. Merging of these axially overlapping clusters during 2D imaging would introduce significant error in quantifying RyR configuration. However, the calibration technique for 3D quantification established here presents an opportunity for accurate examination of RyRs within the cell interior, and to directly compare this organization with that on the cell surface. Schematic 3D representations of the organization of surface and interior RyRs are shown in Fig. 4 *A* and *C*, with selected CRUs presented at two viewing angles (Fig. 4 *B* and *D*). Compared with those from the surface, interior RyR clusters appeared to be more compactly organized along the *z*‐lines. Additionally, the ‘double rows’ of clusters often present at the cell surface were notably absent in the cell interior. Indeed, quantification confirmed that fewer RyR clusters were present between *z*‐lines within the cell interior (Fig. 4 *E*), and that the overall density of RyRs was lower than at the cell surface (Fig. 4 *F*). Nevertheless, the volume of the cell allocated to internal RyRs is much larger than for those at the surface. Thus, we can estimate that internal RyRs constitute roughly 75% of a total of ∼1 million channels in a typical cardiomyocyte (Fig. 4 *G*).

Tight packing of RyRs along *z*‐lines at internal sites was associated with larger RyR clusters than those observed at the cell surface (Fig. 4 *H*, 13.1 ± 1.0 *vs*. 10.1 ± 1.1 RyRs/cluster at cell surface, *P* < 0.05). In addition, close association of neighbouring clusters resulted in the definition of larger CRUs at interior sites, as defined by the number of included RyRs (22.8 ± 2.0 *vs*. 17.5 ± 1.3, Fig. 4 *I*) and clusters (2.3 ± 0.1 *vs*. 1.9 ± 0.1, Fig. 4 *J*), with configurations which were often complex (example in Fig. 4 *D*, zoom‐in of boxed region in Fig. 4 *B*, top‐down and side‐on 3D constructions). Indeed, 10% of internal CRUs contained six or more clusters. However, the most common CRU contained only a single RyR, at both the cell surface and the interior (49% and 50% of CRUs, respectively), suggesting a more prominent presence of ‘rogue RyRs’ than previously appreciated.

**Figure 5:**
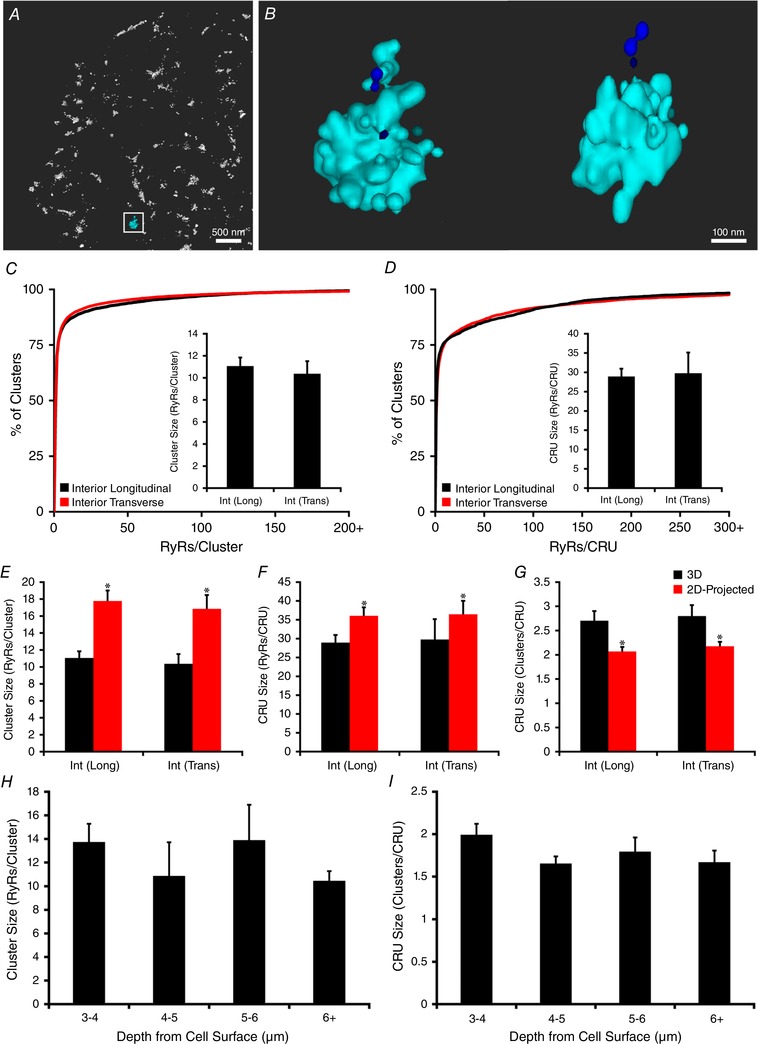
Comparison of 3D interior RyR cluster characteristics in longitudinally and transversely oriented cardiomyocytes Cardiomyocytes were embedded and arranged perpendicularly to allow transverse sectioning, imaging and quantification of RyR organization. A representative 3D rendering of a transversely oriented cell is shown in (*A*), with greater detail of the indicated CRU presented in two orientations (*B*). In comparison with imaging of cells in the longitudinal orientation (Fig. 4), transversely sectioned cells revealed very similar 3D measurements of internal RyR cluster and CRU sizes (*C* and *D*). Projection of these images to 2D resulted in overestimation of both cluster and CRU sizes, regardless of cell orientation (*E* and *F*), and underestimation of the number of clusters/CRU (*G*). *n*_cells_ = 9 longitudinal, 15 transverse from 2 hearts. ^*^ *P* < 0.05 *vs*. 3D measurements. In longitudinally oriented cells, 3D size estimates of RyR clusters (*H*) and CRUs (*I*) were unchanged as the depth of the imaging plane was altered, suggesting rather uniform RyR organization throughout the cell interior. *n*_cells_ = 15, *n*_hearts_ = 4.

Our estimates of internal RyR cluster and CRU sizes are notably smaller than those previously reported by Hou *et al*. using 2D imaging of transversely oriented cardiomyocytes (Hou *et al*. 2015). To investigate whether this discrepancy could be due to imaging of different cellular orientations, we examined cells which were embedded in compound and then stood upright for transverse sectioning. Quantification of 3D RyR cluster and CRU characteristics in these transverse sections was performed using a calibration factor obtained from the surface of plated, longitudinally oriented cells on the same dish. A representative transverse image is presented in Fig. 5 *A*, with a selected CRU shown from two viewing angles in Fig. 5 *B*. Estimated sizes of RyR clusters (Fig. 5 *C*) and CRUs (Fig. 5 *D*) obtained from these images were remarkably similar to those obtained in longitudinal sections. However, 2D projections of internal clusters imaged in either orientation yielded significantly larger clusters and CRUs than 3D representations (Fig. 5 *E* and *F*), and underestimated the number of clusters (Fig. 5 *G*). Thus, superimposition of RyRs within the *z*‐axis likely contributed to large RyR arrangements reported with 2D imaging in previous work.

The similarity of RyR cluster and CRU characteristics determined in longitudinal and transverse planes suggests that RyRs may have rather uniform arrangement across the cell interior. To further investigate this point, we next varied the depth of the imaging plane in longitudinally oriented cells, using four focal points ranging from 3 to 7 μm from the bottom cell surface. Care was taken to exclude the sides of the cell. The outermost of these focal planes, at a depth of 3 μm, was established to contain only internal RyRs since there is a distinct gap between these clusters and those on the cell surface (see Fig. 2 *E*). Mean data show that both RyR cluster (Fig. 5 *H*) and CRU (Fig. 5 *I*) morphology remained similar as the focal plane was moved from the near‐surface to the cell centre.

### Visualization of 3D dyadic geometry

**Figure 6:**
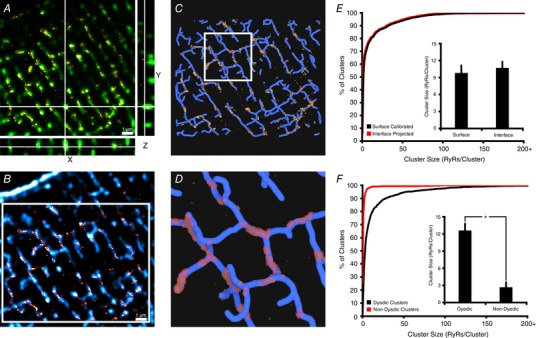
Correlative 3D dSTORM and confocal imaging of RyRs and t‐tubules enables visualization of dyads *A*, RyR images obtained by 3D dSTORM (red) and confocal microscopy (green) were employed to align 3D representations obtained by the two modalities. *B*, a single slice from an aligned 3D stack of RyRs (red, dSTORM) and t‐tubules (blue, confocal Caveolin‐3 + NCX labelling). These data were rendered as a correlative model (see main text), with RyRs packed into the interface of the two signals, assuming a 10 nm dyadic cleft. *C* and *D*, for the boxed region in *B*, a top‐down view of the 3D reconstruction is illustrated in *C*, with greater zoom shown in *D*. RyR positions are represented in yellow, and idealized junctional SR morphology is shown in red. Considerable variability in 3D dyadic configuration is evident, as is a general sparsity of non‐dyadic RyR clusters (15% of all clusters). *E*, the validity of the geometric (interface) model for estimating RyR counts within dyads was assessed by comparing with values based on event‐based calibration (Fig. 2 *B*). As illustrated by cumulative percentage histograms and mean measurements (inset), the two methods provided very similar estimates of RyR cluster size (*n*_cells_ = 4, ^*^ *P* < 0.05). *F*, quantification of non‐dyadic clusters (>250 nm distant from t‐tubules) revealed that these clusters are significantly smaller than their dyadic counterparts, and contain a disproportionate fraction of single ‘rogue’ RyRs. [Correction made on 14 December 2018, after first online publication: Figure 6 was replaced.]

The above results illustrate methods for quantifying RyR distribution within clusters and CRUs in 3D space. However, while these methods provide estimates of RyR numbers, they do not provide insight into the precise orientation of RyRs within these groupings. While the shapes of flat RyR clusters at the cell surface can be estimated by grid‐based fitting (Fig. 1 *C* and *E*), such methods are not possible at internal sites without a reference to indicate the local orientation of the junctional SR. We therefore imaged t‐tubules and RyRs in the same cells to reveal their interface at dyadic junctions. This was done by integrating 3D dSTORM imaging of RyRs (AlexFluor 647) with conventional 3D confocal imaging of t‐tubules (Biotium CF 568). These images were aligned in 3D space, based on RyR images obtained with the two modalities (Fig. 6 *A*), to produce a correlative overlay as shown in Fig. 6 *B* (RyRs shown in red, t‐tubules in blue). For visualization, these correlative data were subsequently rendered as a 3D geometric model, using a fixed t‐tubule diameter of 250 nm (Soeller & Cannell, 1999) and an assumed dyadic cleft of 10 nm to create a junctional SR (jSR) mask. The RyR‐containing portion of this mask was shaped based on its interface with dSTORM‐derived RyR clusters, taking into account their localization error. Representative reconstructed geometries taken from the boxed region in Fig. 6 *B* are shown in Fig. 6 *C* and *D* at progressively increased zoom. Marked variability in dyadic shape and size is readily apparent.

To validate the accuracy of the 3D geometric model for estimating RyR counts within clusters, we compared the prediction of dyadic RyR number by this method with predictions based on counts of RyR blinks (surface‐calibration method, Fig. 2 *B*). Figure 6 *E* shows that the two approaches yielded very similar estimates of RyR cluster sizes, as indicated by mean measurements and the distributions of these values (Fig. 6 *E*). Thus, in addition to providing insight into cluster orientation in 3D space, the interface‐calibration method might serve as an alternative or complementary approach for quantifying dyadic composition. Importantly, such interface‐based calibration techniques could be established for quantification of other proteins for which grid‐based calibration at the cell surface is not possible.

Paired imaging of RyRs and the t‐tubule network additionally enabled distinction between dyadic and non‐dyadic RyR clusters. To this end, non‐dyadic clusters, also known as ‘orphaned’ or ‘rogue’ RyRs (Song *et al*. 2006), were defined as those >250 nm distant from the closest t‐tubule skeleton (Jayasinghe *et al*. 2009). Figure 6 *C* and *D* show that the vast majority of clusters were deemed to be dyadic, while only 14.9 ± 3.7% of clusters were classified as non‐dyadic. Non‐dyadic clusters were also found to be markedly smaller than their dyadic counterparts (Fig. 6 *F*; 2.7 ± 0.9 *vs*. 12.6 ± 1.2 RyRs/cluster, *P* < 0.05).

To provide a greater appreciation of the complexity of RyR organization revealed by these imaging approaches, a representative 3D geometry was animated (Supporting information, Video S1). Variability in dyadic shape and size are readily apparent, as is the sporadic and interspersed positioning of rogue RyRs.

### Comparison of surface and interior calcium spark characteristics

**Figure 7:**
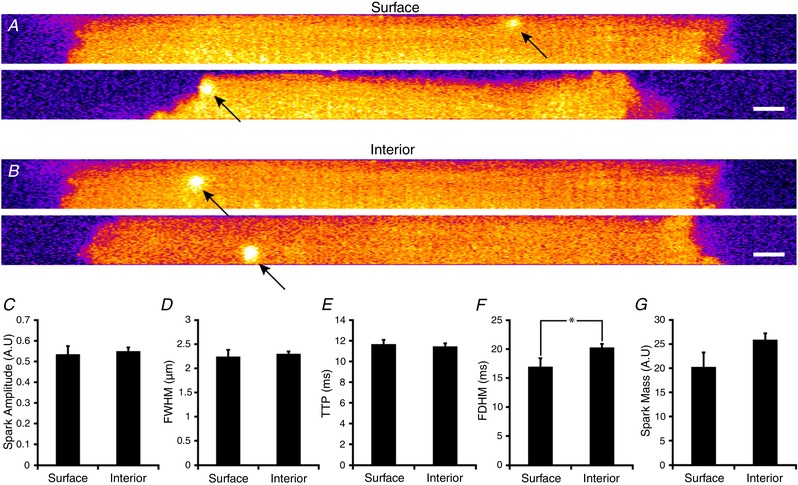
Comparison of Ca^2+^ sparks at the cell surface and interior Rapid time‐series imaging of Ca^2+^ sparks was performed using confocal frames positioned to include both the cardiomyocyte surface and the interior. Representative single frames showing events at these two locations are presented in *A* and *B*, respectively. (Scale bar = 5 μm). Similar amplitude (*C*), full‐width half‐maximum (*D*), and time to peak (*E*) values were measured for Ca^2+^ sparks originating at the surface and interior. However, spark duration (*F*) was significantly longer for internal sparks, and overall spark mass (*G*) tended to be larger. Surface: *n*_sparks_ = 26; interior: *n*_sparks_ = 111; *n*_cells_ = 21, *n*_hearts_ = 3. ^*^ *P* < 0.05 *vs*. surface measurements.

Finally, as our results indicate markedly different organization of RyRs at the cell surface and interior, we examined the consequences for Ca^2+^ sparks originating at the two locations. Rapid time‐series recordings were made of confocal frames positioned to include both the surface and the interior of isolated cardiomyocytes (Fig. 7 *A* and *B*). Sparks originating from the two locations exhibited similar amplitudes, widths and rise times (Fig. 7 *C*–*E*). Spark duration, on the other hand, was longer within the cell interior (25.7 ± 0.4 *vs*. 23.1 ± 0.9 ms, *P* < 0.05, Fig. 7 *F*) and overall spark mass tended to be increased (Fig. 7 *G*), likely reflecting the fact that internal CRUs are larger and more multi‐clustered than surface CRUs (Fig. 4).

## Discussion

In cardiomyocytes, the physiological function of RyRs is critically dependent on their spatial distribution (Walker *et al*. 2014; Macquaide *et al*. 2015). Current understanding of RyR localization has primarily arisen from data obtained by electron microscopy and various forms of 2D super‐resolution light microscopy. While electron microscopy offers unparalleled resolution of individual RyRs, quantification of RyR packing within dyads has proven challenging by these methods (Hayashi *et al*. 2009; Asghari *et al*. 2014). By contrast, and despite being limited by somewhat lower resolution, light microscopy‐based approaches have provided valuable detail regarding RyR arrangement within both clusters and CRUs (Baddeley *et al*. 2009; Hou *et al*. 2015; Macquaide *et al*. 2015; Jayasinghe *et al*. 2018; Kolstad *et al*. 2018) [Correction made on 14 December 2018, after first online publication: Kolstad *et al*. 2018 has been included as a citation for the preceding statement]. However, with conventional 2D super‐resolution imaging, fluorescence is axially summated, leading to errors in quantification for proteins which are vertically overlapping. For this reason, previous work examining RyRs by 2D dSTORM and DNA‐PAINT have been restricted to the surface of cardiomyocytes, where RyR clusters have been assumed to be flatly oriented, without overlap (Baddeley *et al*. 2009; Jayasinghe *et al*. 2018). In the present work we developed techniques for 3D quantification of RyR localization, allowing axial separation of neighbouring clusters. These methods have provided novel insight into the arrangement of RyR clusters and CRUs in 3D space at both the cell surface and the interior, knowledge which is essential for understanding dyadic function. The basis of the developed methodology is exploitation of the observed linear relationship between the number of fluorescence events (blinks) and the number of RyRs (Fig. 2 *B*) (Baddeley *et al*. 2009). By imaging non‐overlapping surface clusters in 3D, we first measured the event numbers for each cluster. RyR counts were then obtained by compressing the 3D stack to a 2D image (Fig. 1 *C* and *E*) and identifying RyR positions using a 30 × 30 nm grid, as described in previous work (Baddeley *et al*. 2009; Hou *et al*. 2015). The slope of this relationship (3.31 events/RyR) was subsequently used as a calibration factor to convert event counts to RyR numbers. This approach enables tallying of RyRs within clusters and CRUs which have irregular protein arrangements, and are not horizontally oriented along the long axis of the cell. Importantly, this method overcomes the poorer axial resolution inherent with dSTORM imaging (median of 64 nm *vs*. 32 nm in the *xy* plane, Fig. 2 *A*), which would have made a volume‐based quantification approach error prone. Despite the lower spatial resolution in the *z*‐axis, our method provided identical estimates for RyR cluster sizes at the bottom and sides of the cell (Fig. 2 *E*). Consistent measurements were also obtained at internal sites regardless of cellular orientation (Fig. 5), suggesting that with suitable calibration, 3D dSTORM can accurately discriminate and quantify RyR clusters which are axially aligned.

### 2D *vs*. 3D surface clusters

Previous work has shown that the surface of cardiomyocytes is not flat, but rather assumes an undulating morphology due to the presence of ridges and grooves surrounding the *z*‐line (Ove Semb *et al*. 1998; Gorelik *et al*. 2006). Thus, it is perhaps not surprising that our 3D imaging at the cell surface revealed an axially variable arrangement of RyRs (Fig. 1 *A* and *B*), which may reflect positioning at different levels of the *z*‐groove. This arrangement resulted in frequent axial overlap of neighbouring protein clusters, indicating that 2D methods for quantifying RyR organization at the cell surface are inherently inaccurate. Indeed, we observed that the calibration factor relating event counts to RyR number was overestimated by >40% when axially overlapping clusters were included in the curve (Fig. 2 *B*, slope = 4.71 *vs*. 3.31 events/RyR). This overestimation of RyR number by 2D imaging also resulted in exaggerated counts of RyRs/cluster, as vertically offset RyRs were erroneously grouped (Figs 1 *C* and *D*, and 2 *C*). Of note, the ‘apparent’ mean 2D‐projected cluster size of ∼12 RyRs/cluster was comparable to that previously reported at the cell surface (13.6 RyRs/cluster (Baddeley *et al*. 2009)), while our present 3D data suggest a value of only ∼10 RyRs/cluster. Furthermore, smaller RyR cluster sizes revealed by 3D imaging included a high fraction of clusters which only contained a single RyR (56% of all clusters), while a markedly lower fraction of these clusters was suggested based on 2D projections (35% in present work; ∼20% in Baddeley *et al*. (2009)).

In addition to incorrect merging of overlapping clusters in 2D projections, we observed that this imaging modality also underestimated the spacing between neighbouring RyR clusters (Figs 1 *E* and *F*, and 2 *D*). This resulted in inappropriate grouping of clusters >100 nm apart into the same CRU (Fig. 3). Using 3D quantification, the average surface CRU was observed to in fact only contain ∼1.9 clusters totalling 17.5 RyRs, a value significantly lower than estimates based on 2D projections in both present and previous work (Baddeley *et al*. 2009). With fewer RyR clusters included in each CRU, 3D analysis also revealed a higher density of surface CRUs than provided by 2D estimates (3.4 *vs*. 2.5 CRUs μm^−3^). Thus, both visual inspection and quantification of 3D dSTORM data revealed rather diffuse organization of RyRs on the cell surface, with the release channels arranged into relatively small clusters which are often located too distant from their neighbours to allow their cooperative grouping into CRUs.

### 3D surface *vs*. interior clusters: functional implications

One major advantage of our current 3D event‐based approach is that it allows for the quantification of interior clusters deeper beneath the cell surface. Although the accuracy of *z*‐localization is depth dependent (Baddeley *et al*. 2011 *a*; Tafteh *et al*. 2016), our calibration method showed consistent estimation of RyR cluster and CRU sizes at internal sites up to a depth of ∼6 μm (Fig. 5 *H* and *I*). Thus, the developed method allowed direct comparison of RyR organization at the cell surface and interior by varying the focal plane within the cell. Interior clusters were observed to be organized in a more ‘orderly’ manner along *z*‐lines compared to those on the surface, where more clusters were found in between *z*‐lines (Fig. 4 *A*, *C* and *E*). Additionally, interior clusters lacked the ‘double row’ arrangement of RyRs that is often apparent at the cell periphery (Chen‐Izu *et al*. 2006; Jayasinghe *et al*. 2009), including in live‐imaged cardiomyocytes (Hiess *et al*. 2018). Tighter packing of RyRs along *z*‐lines at internal sites was associated with larger cluster sizes and more clusters being included in the average CRU (Fig. 4 *I* and *J*). Thus, the overall structure of CRUs at internal sites showed considerably greater complexity than their surface counterparts, as they were often multi‐clustered and, as illustrated by correlative imaging (Fig. 6), irregularly shaped in 3D space to create functional couplings with t‐tubules.

Our observation that RyR clusters and CRUs are larger at internal sites than the cell surface is in keeping with a previous study by Hou *et al*. (2015). However, their data for internal sites showed much higher mean counts of RyRs per cluster and CRU (63 and 103, respectively) than we observed in the present study (13 RyRs/cluster, 23 RyRs/CRU). Hou *et al*. also observed some very large clusters containing many hundreds of RyRs within internal sites; these were not observed in the present study. Our data show that this discrepancy did not result from differences in imaging orientation, as very similar RyR cluster and CRU characterstics were obtained from cells positioned longitudinally or sectioned transversely (Fig. 5). Rather, our results suggest that 2D imaging employed in the previous work likely inflated RyR counts as RyR clusters positioned around the same t‐tubule were superimposed (Fig. 5 *E* and *F*).

Our estimates of CRU size are also lower than those based on EM tomography of dyads in mouse cardiomyocytes, which indicated that an average size dyad could hold up to 43 RyRs (Hayashi *et al*. 2009). This estimate was, however, dependent on tight RyR packing within the dyad, and does not take into account the complex allocation of RyRs between clusters presently observed. Overall, we calculated an RyR density of ∼57 channels μm^−3^ in the cardiomyocyte, which is in general agreement with a previous estimate based on ryanodine binding rather than imaging (Bers, 2001).

Our results have important implications for understanding both spark‐ and non‐spark‐mediated Ca^2+^ release. We show that CRUs are smaller than previously reported, both at peripheral and internal sites, containing an average of only 18 and 23 RyRs, respectively. Importantly, these values are roughly in line with previous work which estimated that typical Ca^2+^ sparks within the cell interior result from the opening of ∼20–30 RyRs (Shkryl *et al*. 2012). Thus, Ca^2+^ sparks may reflect the coordinated openings of all RyRs in averaged‐sized CRUs, an interpretation which contrasts with that of previous work reporting much larger CRU sizes from 2D imaging (Hou *et al*. 2015). However, while the average CRU size may be sufficient to generate the average detected Ca^2+^ spark, very small CRUs are the most common. In fact, we observed that single RyR CRUs comprised half of all CRUs (49% of surface CRUs and 50% of interior CRUs), but a full 90% of the non‐dyadic RyR fraction (Fig. 6 *F*) [Corrections added on 14 December 2018, after first online publication: The preceding statement refers to Fig. 6*F* in this version]. Some of these single RyRs could be part of a trafficking pool of channels which are *en route* to their final positions in the SR. However, previous work has suggested that single ‘rogue’ RyRs are functional (Sobie *et al*. 2006). Ca^2+^ release from these RyRs is thought to be undetectable by standard methods for measuring Ca^2+^‐dependent fluorescence, giving rise to ‘invisible’ or ‘silent’ SR Ca^2+^ leak (Sobie *et al*. 2006). Our findings suggest that non‐dyadic RyRs may be particularly important contributors to such leak. Single or small groups of RyRs have also been implicated in ‘quarky’ SR Ca^2+^ release events, a phenomenon detectable only by careful simultaneous measurement of cytosolic and SR Ca^2+^ levels (Lipp & Niggli, 1998; Brochet *et al*. 2011). Interestingly, quarky release has been proposed to result from the opening of a subset of RyRs within a larger CRU, which fits with our present observation that internal CRUs often exhibit complex, multi‐clustered configurations. Silent, non‐spark‐mediated Ca^2+^ leak in cardiomyocytes, which is a large contributor to overall Ca^2+^ leak (Zima *et al*. 2010), may therefore be linked to both a high proportion of small CRUs and larger, multi‐cluster CRUs.

While the majority of previous experimental work has examined Ca^2+^ sparks only within cardiomyocytes using confocal microscopy, our results provide new insight into differing spark characteristics at the surface and interior, and how this relates to CRU configuration. We observed that although interior CRUs contain a greater number of RyRs than those on the surface, Ca^2+^ spark amplitudes were similar at the two locations. This finding is in keeping with previous computational modelling, which has shown that Ca^2+^ spark amplitude is relatively insensitive to RyR numbers when the CRU size is ∼9 or higher (Cannell *et al*. 2013). However, interior Ca^2+^ sparks were prolonged compared to those on the cell surface (Fig. 7 *F*), in agreement with work showing that spark duration increases in concert with RyR number (Sato *et al*. 2016), as greater [Ca^2+^] in the jSR increases channel open time and delays the onset of spark termination (Williams *et al*. 2011; Sato *et al*. 2016). In terms of overall Ca^2+^ leak, it might be predicted that internal RyRs are greater contributors since these channels constitute approximately 75% of the roughly 1 million channels found in an average cardiomyocytes (Fig. 4 *G*). On the other hand, smaller surface clusters would be predicted to exhibit higher channel open probability (Sobie *et al*. 2006). Differing arrangements of RyRs may also have functional implications for the generation of Ca^2+^ waves. The broader distribution of surface CRUs away from *z*‐lines results in shorter distances between neighbours, which previous work has shown may promote regenerative wave propagation (Izu *et al*. 2013). Thus, it is plausible that Ca^2+^ waves could spread preferentially along the cell surface. Dispersion of internal clusters during pathology may similarly augment wave propagation within the cell interior, as recently reported in atrial fibrillation (Macquaide *et al*. 2015) and heart failure (Kolstad *et al*. 2018).

### Insights from correlative RyR and t‐tubule imaging

While the presented calibration method provides an opportunity for estimating the number of RyRs within clusters and CRUs, it does not yield information regarding the orientation of RyRs within these groupings. To this end, we combined RyRs imaged with 3D dSTORM and t‐tubules imaged with confocal microscopy to estimate the arrangement of RyRs within dyadic junctions at the interface between the two images (Fig. 6). In addition to enabling dyadic visualization of the complexity and variability of dyadic organization, we expect that the geometries obtained by this technique will aid 3D mathematical modelling of the dyad. Indeed, previous modelling work has shown that the specific placement of RyRs relative to dyadic membranes crucially determines Ca^2+^ spark characteristics (Cannell & Soeller, 1997; Tanskanen *et al*. 2007; Cannell *et al*. 2013). The geometric model additionally provides an opportunity to distinguish between dyadic and non‐dyadic RyRs. These analyses indicated that approximately 15% of all clusters are non‐dyadic, an estimate that is in agreement with the 16% reported previously based on confocal data (Jayasinghe *et al*. 2009). Further examination of these distinct populations revealed that non‐dyadic RyRs are present in very small clusters (Fig. 6 *F*) [Corrections added on 14 December 2018, after first online publication: The preceding statement refers to Fig. 6*F* in this version].

We observed that the projected RyR number within the dyadic interface quantitatively resembled estimates based on our surface‐based calibration method (Fig. 6 *E*). Thus, this type of correlative imaging may be an alternative strategy for estimating RyR number in tissue sections or other applications for which surface data are not readily available. Our current approach relied on confocal imaging of t‐tubules, which has inherently lower resolution than dSTORM images, but allowed smooth, continuous rendering of t‐tubule surfaces. Nevertheless, though our current model is compatible with the data, it is not the only possible one, as the low confocal resolution leads to a non‐uniqueness problem. Thus heuristic rules (e.g. defining t‐tubule radius, cleft space distance, etc.) are required for 3D reconstruction. While we expect that dual colour 3D dSTORM imaging could provide greater accuracy for interface‐based quantification, this approach is technically challenging. Although others have had success with this technique in non‐cardiac cells (Jones *et al*. 2011; Baddeley *et al*. 2011 *b*), high blinking efficiency of both fluorophores is necessary to effectively render 3D localizations with adequate precision. While Alexa Fluor 647 is sufficient for these purposes, we have yet to find another secondary antibody with similar efficacy under the same experimental conditions. Recent work by Crossman *et al*. demonstrated the suitability of using wheat germ agglutinin (WGA) with 2D dSTORM in identifying the human t‐tubule network (Crossman *et al*. 2017). One advantage of using WGA is that it labels the entire cellular membrane, revealing parts of the t‐tubule network that may be missed when using our current Cav‐3/NCX1‐based approach. However, in our experience WGA does not consistently label t‐tubules in rodents. Alternatively, 3D dSTORM images of RyRs might be combined with images of t‐tubule and SR membranes obtained by EM tomography, an approach referred to as correlative light electron microscopy (de Boer *et al*. 2015). However, it should be noted that while the resolution of EM techniques is unsurpassed, 3D quantification of RyRs within the arching jSR is challenging and labour intensive. Thus we suggest that such techniques provide complementary information to our present 3D dSTORM imaging approaches, which provide localization information for thousands of RyR clusters.

### Applicability to 3D quantification of other proteins

We anticipate that the developed techniques for estimating RyR numbers and arrangements in 3D space may also be applied to other proteins. In cardiomyocytes, several other dyadic proteins have been demonstrated to display clustering properties, including junctophilin‐2 (Jayasinghe *et al*. 2018), L‐type Ca^2+^ channels (Scriven *et al*. 2010) and the sodium–calcium exchanger (Wang *et al*. 2014). With knowledge of the associated packing density, quantification of proteins clusters within the cell interior could theoretically be estimated by the dyadic interface method (Fig. 6), or by establishing a calibration curve on the cell surface relating the number of fluorescence blinks to number of proteins (Fig. 2). Indeed, for monomeric proteins, quantification would be simplified by the fact that blinking events in close proximity could be more easily attributed to neighbouring proteins. Such assumptions are more problematic for the tetrameric RyR, as several subunits are simultaneously labelled by antibodies (Jayasinghe *et al*. 2018). On the other hand, RyR tallying on the cell surface is abetted by the fact that the protein is of similar size to the presently attained *xy* resolution. Therefore, quantification of smaller proteins would likely benefit from higher resolution imaging techniques such as DNA‐PAINT (Jayasinghe *et al*. 2018). Alternatively, photoactivatable fluorophores may be employed to estimate protein number, although previous work has shown these fluorophores also require suitable calibration techniques (Lee *et al*. 2012). Future work may aim to employ these or related techniques such as SNAP tagging to examine 3D protein arrangement in live cells.

### Limitations

Several possible limitations should be noted regarding our 3D RyR imaging approaches. The calibration technique employed for estimating RyR number is based on the assumption that RyRs on the cell surface are fully packed in a crystalline array (Yin & Lai, 2000). While this same assumption has been made in previous dSTORM studies (Baddeley *et al*. 2009), the lateral resolution of the technique is only roughly equivalent to the dimensions of a single RyR. Therefore, the packing of RyRs within the obtained 2D geometry (Fig. 1 *C*), and thus our calibration factor, are likely somewhat overestimated. Indeed, recent EM studies have shown that in fixed ventricular myocytes, RyR packing is non‐uniform, revealing unfilled spaces within clusters (Hayashi *et al*. 2009; Asghari *et al*. 2014). Thus, while our 3D quantification showed a smaller number of RyRs within each cluster and CRU than estimated from 2D imaging (Fig. 2 *C* and 3 *E*), their true size may be even smaller. However, due to the lower resolution in the axial plane, the real number of clusters in each CRU may be *larger*, since a subset of vertically overlapping clusters likely remains unresolved. Finally, as our present work has been performed on isolated and fixed cardiomyocytes, we cannot rule out that these procedures alter RyR configuration artefactually. Ultimately, our current approximations of RyR organization should be verified in future work using cryoEM approaches in intact tissue at the highest resolution possible.

### Conclusion

In summary, we have employed dSTORM with PRILM technology to develop novel, quantitative methods for assessing 3D RyR organization within cardiomyocytes. 3D imaging revealed that RyR clusters at the cell surface frequently overlap in the axial plane, leading to their erroneous merging by conventional 2D imaging modalities. By selecting non‐overlapping clusters, we created a calibration curve for estimating the number of RyRs based on recorded fluorescence blinks. Examinations performed at both the cell surface and interior revealed smaller RyR clusters than previously reported, and smaller functional groupings of RyRs into CRUs. We observed that CRUs at internal sites are larger and more complex than those at the cell surface, but that nearly half of all CRUs contain only a single ‘rogue’ RyR. We further demonstrated that 3D dSTORM imaging of RyRs can be combined with 3D confocal microscopy of t‐tubules to enable visualization and quantification of RyRs within dyadic junctions. Finally, we observed that differences in the arrangement of CRUs at the cell surface and interior have distinct functional consequences, as the larger and more complex internal CRUs generate longer duration Ca^2+^ sparks. We anticipate that similar methods can be applied to study the nanoscale arrangement and function of diverse proteins in both cardiomyocytes and non‐cardiac cells.

## Additional information

### Competing interests

None declared.

### Author contributions

X.S., A.G.E., C.S. and W.L. were responsible for the conception and design of the study. X.S. carried out all imaging studies, J.v.d.B, Y.H. and N.M. optimized the script used for image reconstruction. X.S., C.L. and T.R.K. carried out image analysis. X.S. and J.v.d.B. rendered the 3D schematics. D.C. and P.M.K‐H. designed custom software and performed analysis of Ca^2+^ sparks. C.C. designed and verified the custom NCX antibody. X.S. wrote the paper with input from all authors. All authors have read and approved the final version of this manuscript and agree to be accountable for all aspects of the work in ensuring that questions related to the accuracy or integrity of any part of the work are appropriately investigated and resolved. All persons designated as authors qualify for authorship, and all those who qualify for authorship are listed.

### Funding

This work was supported by the European Union's Horizon 2020 research and innovation programme (Consolidator grant, W.E.L.) under grant agreement No. 647714. Additional support was provided by The South‐Eastern Norway Regional Health Authority, Anders Jahre's Fund for the Promotion of Science, The Norwegian Institute of Public Health, Oslo University Hospital Ullevål, and the University of Oslo. Research reported in this publication was supported by the Maximizing Investigators' Research Award (MIRA) (R35) from the National Institute of General Medical Sciences (NIGMS) of the National Institutes of Health (NIH) under grant number R35GM124977 (to P.K.H.).

## Supporting information


**Video S1**. Artistically rendered video of the T‐tubular and RyR network based on realistic geometries.
